# “It’s the Attraction of Winning That Draws You in”—A Qualitative Investigation of Reasons and Facilitators for Videogame Loot Box Engagement in UK Gamers

**DOI:** 10.3390/jcm10102103

**Published:** 2021-05-13

**Authors:** Laura Louise Nicklin, Stuart Gordon Spicer, James Close, Jonathan Parke, Oliver Smith, Thomas Raymen, Helen Lloyd, Joanne Lloyd

**Affiliations:** 1 Cyberpsychology Research Group, Department of Psychology, Faculty of Education, Health and Wellbeing, MC323, University of Wolverhampton, Wolverhampton WV1 1LY, UK; Laura.Nicklin@wlv.ac.uk; 2 School of Psychology, Faculty of Health, University of Plymouth, Plymouth, Devon PL4 8AA, UK; Stuart.Spicer@plymouth.ac.uk (S.G.S.); james.close@plymouth.ac.uk (J.C.); Helen.Lloyd-1@plymouth.ac.uk (H.L.); 3 Sophro LTD-Gambling Research and Education, Newark Beacon Innovation Centre, Cafferata Way, Newark NG24 2TN, UK; Jonathan@sophro.uk.com; 4 School of Law, Criminology and Government, Faculty of Arts, Humanities and Business, 112, 19 Portland Villas, University of Plymouth, Plymouth Devon PL4 8AA, UK; Oliver.Smith@plymouth.ac.uk; 5 Department of Social Sciences, Faculty of Arts, Design and Social Sciences, 223 Lipman Building, Northumbria University, Newcastle upon Tyne NE1 8ST, UK; Thomas.Raymen@northumbria.ac.uk

**Keywords:** loot boxes, video-gaming, motivations, motives, microtransactions, gambling, addiction, qualitative, monetisation

## Abstract

Excessive engagement with (increasingly prevalent) loot boxes within games has consistently been linked with disordered gambling and/or gaming. The importance of recognising and managing potential risks associated with loot box involvement means understanding contributing factors is a pressing research priority. Given that motivations for gaming and gambling have been informative in understanding risky engagement with those behaviours, this qualitative study investigated motivations for buying loot boxes, through in-depth interviews with 28 gamers from across the UK. A reflexive thematic analysis categorised reasons for buying into seven “themes”; opening experience; value of box contents; game-related elements; social influences; emotive/impulsive influences; fear of missing out; triggers/facilitators. These themes are described in detail and discussed in relation to the existing literature and motivation theories. This study contributes to understanding ways in which digital items within loot boxes can be highly valued by purchasers, informing the debate around parallels with gambling. Findings that certain motivations were disproportionately endorsed by participants with symptoms of problematic gambling has potential implications for policy and warrants further study.

## 1. Introduction

Loot boxes are chance-based in-game purchases prevalent within video games (almost a billion of which are currently ranked as suitable for children [1]). These boxes are most commonly purchased through real world currency transactions, where money is paid to open a virtual “pack”, “chest”, or similar, though they can also be obtained in-game through continuous play to earn in-game credits at a slower rate. Comparisons have been drawn with gambling [2,3] because box contents vary in value, be it perceived/psychological worth (often linked to rarity), objective price (evident where they are available to buy outright), or “resale” value, within third-party markets, and the pattern of “rewards” (i.e., highly desirable contents being revealed) typically follows a variable reinforcement ratio, that is also characteristic of gambling activities. Furthermore, they are widely engaged with, attracting revenues exceeding those generated from some forms of gambling [4]. While structural and psychological similarities to gambling [2,3] have led several jurisdictions—including The Netherlands, China, Australia and Singapore—to introduce legislation on loot boxes, with some countries (e.g., Belgium) even banning them, they are not, at the time of writing, covered in the UK by the 2005 Gambling Act, due to the lack of perceived monetary value of potential winnings.

Nonetheless, loot boxes continue to receive attention from UK policymakers, academics, and the general public [2,5], with media reports highlighting consequences of compulsive purchasing, and accumulating evidence of links between loot box expenditure and problem gambling [4,6]. In July 2020, the House of Lords called for their regulation as gambling, and a parliamentary petition called for the UK government to “Extend the Gambling Act to cover Loot Boxes” [7], followed by a call for evidence by the Department of Digital Culture, Media and Sport [8]. Further adding to concerns, links have also been identified between problematic engagement with loot boxes and symptoms of disordered gaming [4].

However, unless loot boxes are outlawed—unlikely within the UK and other jurisdictions where gambling is legal—continued research is required to better understand factors associated with problematic engagement and expenditure. Given the psychological similitudes to gambling [2], factors that have been important in understanding gambling behaviour and associated risks may be relevant. For example, Brooks and Clarke [9], found that distorted perceptions of chance and probability, which are associated with disordered gambling [10], also correlated with “risky” loot box engagement.

Motivation research has contributed significantly to understanding of gambling behaviour [11] and could therefore enhance our understanding about motivations for loot box purchasing. Traditional gambling motivation is multidimensional, with individual and societal drivers [10]. Commonly-identified subscales or factors include social and fun/excitement [11,12,13,14], coping/escape [12,13,14], and money [12,13,14]. Additional motivations such as intellectual challenge, leisure-based identities, and recreation/time-filling have also been identified [15,16]. Individual differences in motivations have been identified [16], and some (such as “escape” and “mood modification”) are more strongly associated than others (such as “fun”) with the risk of problematic gambling, comorbid mood disorders, and other addictive behaviours e.g., drinking and substance use [17]. Thus, it is likely that loot box motivations are similarly variable, with different motives associated with greater or lesser risk of harm.

Before this can be explored systematically and quantifiably, it is necessary to establish the full range of loot box motivations, which despite similarities with gambling, also diverge. Whereas the “prize”, in gambling, is typically money, in loot boxes it is digital content, whose value is variable, subjective, and entangled with factors such as involvement in the videogame [18,19]. Furthermore, gaming is not a homogeneous activity—people engage through a variety of platforms (e.g., mobile, console, and personal computer), in a variety of play modes (collaborative, competitive, and individual), and styles, with a wide array of game genres [20]. Thus, while some motives for opening loot boxes might mirror gambling motivations, others may echo gaming motivations, or represent an amalgamation of the two. Existing gaming motivations scales include the Gaming Motivation Scale [21], with subscales relating to “intrinsic” and “extrinsic” drivers, which are linked to self-determination theory [22] and the idea that actions are motivated by need satisfaction [20]. The value of studying gaming motivations has also been demonstrated, as they can predict gaming involvement, and particular factors are differentially associated with wellbeing measures such as anxiety and depression [21,23].

The only study to have specifically explored people’s motivations for loot box engagement found that some (e.g., fun and excitement) paralleled those for gambling, whereas others (e.g., the desire for gameplay advantages and/or to collect particular items) were distinct [24]. This online survey comprised predominantly male 16–18 year olds, recruited via an online forum, with only brief responses (“utterances”) collected from a single free-text box grouped into broad categories. Thus, whilst academic research into gaming and gambling motivations is mature, there is a paucity of evidence around factors driving loot box engagement—despite significant attention amongst policymakers, the public, and academics [25], and the fact that they are increasingly popular, lucrative, and focal to developers’ business models. The current study utilised in-depth semi-structured interviews with a diverse UK-wide sample to contribute a rich, novel understanding of the nuanced factors driving this behaviour. Our research question was broad: “why do people buy and open loot boxes?” We aimed to discover, from the perspective of individuals with lived experience, how and why different factors motivate them.

## 2. Materials and Methods

### 2.1. Data Collection

One-to-one, semi-structured qualitative interviews (lasting from 35 to 68 min) were administered remotely (due to COVID-19) via telephone or online (using WhatsApp, Skype, Discord, Zoom or Microsoft Teams) and recorded.

As part of our “trustworthiness protocol” [26] (p. 123) to ensure rigor, and maximise “credibility, transferability, dependability, and confirmability” [27], interviewers L.L.N. and S.G.S. engaged in peer debriefing after conducting mock interviews, and after the first few research interviews—enabling reflection on style, technique, accuracy, and practice.

### 2.2. Participants

Twenty-eight gamers (19 males, 9 females, mean age 28.9 (range 18–56)) who played at least one game on any platform, including mobile, had purchased at least one loot box, and were aged 16+, were recruited purposively (for a diverse demographic and geographic range) from England, Scotland, Wales and Northern Ireland via online posters, emails, targeted social media posts, and snowball sampling. Pseudonyms are presented for anonymity.

### 2.3. Instruments and Procedure

Upon registering interest, participants were provided with an information sheet, consent form and demographic questionnaire (age; gender; ethnicity; geography; marital, living, occupational, individual salary, and educational status), via QualtricsXM survey software (Manufactured by Qualtrics, Seattle, WA, USA).

Semi-structured qualitative interviews utilised a topic guide, refined (for content and language) via workshops attended by stakeholders with personal and/or professional experience of loot box engagement. Interviews covered: introductions and “warm up” questions; general gaming (e.g., “what kind of gaming do you do?”); loot box questions (e.g., “what makes you decide to buy a loot box?”) and additional questions (about gaming during COVID-19; streaming, monthly, yearly and all time loot box expenditure, and suggestions for further research) not reported here. Participants then completed the Problem Gambling Severity Index (PGSI) [28], and the Internet Gaming Disorder Scale IGD-SF9 [29], in order to provide a detailed picture of the composition of the sample, and whether they were experiencing any symptoms of problematic engagement with gaming and/or gambling. The PGSI is a 9-item, well-validated measure of problematic gambling, with response options of “never” (scored 0), “sometimes” (scored 1), “often” (scored 2), and “almost always” (scored 3), giving a total score between 0 and 27, with scores of 3–7 indicating moderate-risk gambling and 8 or over indicating problem gambler status [28]. The IGD is a 9-item, well-validated measure of problematic gaming, scored on a 5-point Likert-scale, with a possible range from 9 to 45, with higher scores indicating more severe problems, and a score of 32 or above used to indicate “disordered gaming” [30]. Table 1 summarises sample demographic characteristics, PGSI and IGD scores, and loot box expenditure. Participants received a GBP 15 shopping voucher in recompense for participation. “x” refers to an answer not known or given.

### 2.4. Analytical Process

Interviews were conducted by L.L.N. (*n* = 14), S.G.S. (*n* = 13) and T.R. (*n* = 1), transcribed verbatim, and imported into NVIVO 12 (Manufactured by QSR, Melbourne, Australia). To support credibility, transferability and dependability, researchers utilised journaling and notation throughout data collection, coding, streamlining, theming and analysis. Reflexive thematic analysis [31] was carried out, following Braun and Clarke’s six steps [32], and was wholly inductive, with themes dictated by the data. Steps 1–3 (familiarisation with the data, coding, and generation of initial themes) were conducted by the researcher who collected the data, but 6 transcripts were exchanged between L.L.N. and S.G.S., and coded inductively and naively for comparison; to ascertain clarity, consistency and ensure outcomes were fully explored and extracted. J.L. audited data collection, coding and streamlining choices, and O.S. and H.L. blind-coded 3 transcripts to enhance trustworthiness.

Steps 4–6 (reviewing, defining and naming the themes) occurred through several full-team discussions, to ensure all themes were supported by the data and none were overlooked, and checked for coherence and consistency. The multidisciplinary research team’s varying epistemological and ontological stances were respected, and we followed principles of consensual qualitative analysis [33]. All themes were agreed upon and retained, and L.L.N. then conducted a line-by-line reading of all transcripts to ensure all codes were assigned to the most appropriate theme.

### 2.5. Ethics

Ethical approval was granted by Research Ethics Committees at University of Wolverhampton (Approval Code: 143208) and Plymouth University (Approval code: 19/20-1219), and BPS Ethical Principles were followed throughout.

## 3. Results

### 3.1. Participants

Table 1 summarises sample demographic characteristics, PGSI and IGD scores, and loot box expenditure. Most participants reported playing on multiple platforms (24/28 played at least two of console, mobile and personal computer (PC), and most engaged in multiple play styles, but some specialised in co-operative, competitive, or solo modes. We did not collect checklist data on games or genres played, but within interviews, participants reported a diverse range of game genre preferences, encompassing sports-based competitive games (e.g., “FIFA”); first-person and team shooter games (e.g., “Call of Duty”); role-play games (e.g., “Grand Theft Auto”); Massively Multiplayer Online Role Playing Games (MMORPGs) (e.g., “World of Warcraft”); Battle Royale (e.g., “Fortnite”), and mobile games (e.g., “Candy Crush”).

### 3.2. Loot Box Motivators—Thematic Analysis

**Figure 1:**
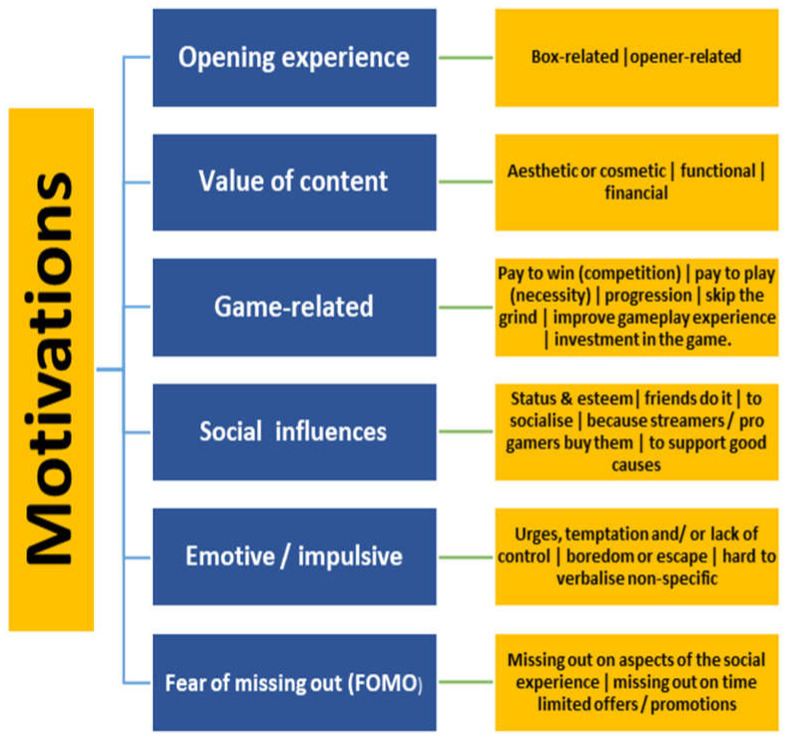
Motivations related to themes and subthemes identified from qualitative interviews.

Through inductive thematic analysis, we categorised nuanced, interconnected factors that influenced loot box engagement into seven overarching themes (with subthemes). These are summarised in Figure 1, below, with illustrative quotations in Table 2 and numbers of participants who spoke of each theme presented in Table 3 and Table 4; but as caution is required in interpreting numbers as indicators of prevalence/significance [34] we utilised the terms “some” (1–8 participants), “many” (9–18 participants), and “most” (19–28 participants). As we present each theme, we highlight any notable features of the participants who endorsed that theme, e.g., if it seemed to be particularly frequently referred to by a certain demographic, or by those scoring above threshold for problematic gambling and/or gaming. Where we do not mention any such effect, it can be assumed that we noted no striking patterns. We restricted these observations to differences that were particularly salient, to avoid the risk of overstating the importance of random variation within a small sample.

### 3.3. Opening Experience

The rewarding nature of opening the loot box directly motivated many participants, and this theme encompassed both features of the box-opening and the feelings that this evoked.

Many loot boxes provided a drawn-out opening experience with exciting visual, auditory and haptic feedback, which most participants described finding “exciting”/“thrilling”/“entertaining” and, thus, motivating. Some described how games use a theatrical “reveal” with teasers to capitalise on this (“it’s very drawn out, it’s very theatrical, it’s ‘Oh my god, psssh, here’s your card, psssh…OH MY GOD, IT’S RONALDO…oh, no it’s some bum’” (Oscar)). These simulated “near misses” were described as particularly engaging, and some highlighted an addictive quality in their reaction to them (“the way the animation played out…like, “ooh, I wonder who I got there”…you just, sort of, got addicted to that” (Sharon)), demonstrating the link between features of the opening experience and the opener’s reactions.

Some participants reported pleasure from simply opening the box, but enjoyment of a sense of “winning”/“success” upon opening, and excitement at discovering what is inside (“it’s just exciting…to see if it’s what you wanted” (Susan)) suggest that the enjoyment of opening the box is difficult to separate from the contents and their value (discussed under the next theme).

Most reported thrill and excitement (or a “buzz”/“rush”) when opening boxes, due to the anticipation of winning something good—resonating with language often used in relation to gambling. Some described the excitement as fleeting, with a cycle of tension and release between purchases and one remarked on having “that pent up wait, like… aggression of not having enough money to buy them” followed by “excitement of realising I had the money to buy them.” (Neil).

The potential for the box to contain something of value was, for almost everyone, crucial to generating excitement, although one individual (a frequent, cross-platform multistyle/genre gamer) enjoyed the opening experience in itself (even enjoying a loot box simulator with no bearing on gameplay: “you don’t actually spend money but…even just doing that…was thrilling” (Susan)). Susan’s preference for cosmetic items and lack of explicit social motivations may partially explain why she enjoyed opening boxes even when she did not get the items inside, as she did not “need” them to progress or impress. Interestingly, despite acknowledging the “thrill” of opening as a motivator, and having gambled in the past, she scored zero on the PGSI (had no problem gambling symptoms), and explicitly valued the ability to obtain excitement and “fulfilment”—“without spending money”.

### 3.4. Value of Box Contents

Almost unanimously, participants opened boxes because they wanted to know what was inside, but beyond curiosity, they wanted to see if it would be valuable, as value of contents varied considerably, in the ways described below.

#### 3.4.1. Financial Value

Some were motivated to purchase loot boxes for the opportunity to win something worth more than the cost of the box. Often, items obtainable within a loot box can also be bought outright, so if a skin costing GBP 30 in an in-game shop is found inside a GBP 5 loot box, the individual gains something of superior financial value. Several gamers described this as a motivation—particularly when they could not afford to purchase a desired item outright.

Some referenced the idea that time is a (financially) valuable commodity that can be saved by buying loot boxes, and described weighing the cost of loot boxes against the time it would take to earn them through gameplay (where they can be “earned” via in-game labour (“grinding”)), or referenced how purchasing a loot box containing functional items could save them what they described as “valuable time” (compared with slower progression through gameplay). While these participants were diverse in their other motivations and most of their demographics, they shared the fact that they were all low/relatively low earners (see Table 1, which may have heightened sensitivity to financial value.

Some referenced the potential to acquire sought-after items which could be sold on (at profit) for gaming or real world currencies, with some even trading loot box contents according to fluctuating value on secondary markets (where people sell digital items for real world currencies external to the source game). While some described decreased opportunities for this (due to gaming companies’ restrictions), some described “workarounds” such as equipping a gaming account with “valuable” items before selling it on. Everyone who highlighted this type of financial motivation spoke of several other motivations, i.e., none bought loot boxes solely for financial gain, and they tended to be amongst the highest spenders on loot boxes within our sample.

#### 3.4.2. Aesthetic or Cosmetic Items

Most participants were motivated by perceived value of aesthetic or cosmetic items (i.e., those with no functional benefit to gameplay or performance); some of which are only available from loot boxes. These skins, costumes, colourful or patterned versions of items, and character dances/animations, held considerable value for many participants. Some simply “liked the look of” the items, and/or felt they enhanced their avatar’s appearance (“skins are the main one, if I want to look good.” (Ian)). Cosmetic appeal was, for some, based on personal taste (e.g., linked to a movie they enjoyed), but for many it was linked to rare, sought-after or trendy skins, which attract attention due to their rarity and, by extension, value. Many described how social desirability influenced the value of aesthetic items (discussed later, within social factors), where, for example, skins obtained from loot boxes provided in-game status, and guarded against appearing to be a “default” or a “noob” (i.e., someone new to the game, presumed to lack skill).

#### 3.4.3. Functional Items

Functional items such as a superior guns, vehicles, tools or armour (and by extension, loot boxes potentially housing them), were valued by many participants for their impact upon performance and/or progression, i.e., because they enhance chance of success or progression (against others, or within the game). Some saw value only in functional items (“unless it affects the game-play, I don’t really need it” (Harry)).

For players of competitive games such as first-person shooters, sports, and driving-based games, the value of a functional item was heavily connected to its ability to increase the chances of beating others. In other game genres, particularly mobile games, functional loot boxes were often sought-after because their contents aided progression or continued engagement with the game—particularly when the difficulty level outmatched their skill level.

Most participants valued both cosmetic and functional items, but there was typically a preference for one or the other. Those who valued functional, but not cosmetic items tended to view the latter as pointless but harmless, whereas those who valued cosmetic, but not functional items often expressed disapproval of their existence and the concept of “pay to win”, via “boosting” or “cheating” (“boosting your way through a game; I don’t see the point.” (Mia)). The small number of participants who told us that they solely valued functional-item loot boxes were predominantly males over 30 who played multiple styles of games on multiple platforms (i.e., were relatively ‘hardcore’ gamers). To better-understand factors motivating functional-item loot box purchasing, it is necessary to consider how they impact upon gameplay, alongside purchasers’ broader gaming motivations.

### 3.5. Game-Related Elements

#### 3.5.1. Progression

Many spoke of buying loot boxes because their contents facilitated in-game progression, through features such as extra lives, time-savers or “skips” past sticking points (“I’d do it when I’m just fed up and stuck” (Kate))—prevalently mobile games; almost all of these gamers played mobile games, and solo gaming, amongst other types. Some reflected how in-game mechanics like “pinch points” interact with their desire to overcome obstacles and continue gaming, resulting in the (sometimes reluctant) decision to purchase a loot box.

#### 3.5.2. Skip the Grind

Some described buying loot boxes to “skip the grind”; another progression-related motive, involving a shortcut past tedious, rather than difficult, content. Conversely, loot boxes can be obtained in some games through grinding, but some described how the high time investment encouraged them to spend money instead (“I could buy some FIFA points here like for GBP 6 rather than me grind for two hours to open one of these packs” (Oscar)).

#### 3.5.3. “Pay to Win”

Some participants described how functional-item containing loot boxes presented a means of “paying to win”, because buying enough boxes can potentially yield items facilitating competitive success, without the player necessarily having to develop skills. While this practice was looked down upon by some, others were attracted to it, and willing to pay for an advantage; all of the latter reported that loot boxes enhanced their gameplay and that they found opening loot boxes exciting, but these features were shared by many other participants, and there were no pronounced distinguishing characteristics about this group.

#### 3.5.4. “Pay to Play”

Many participants were driven to purchase functional-item loot boxes not to seek competitive advantage, but because the practice was so widespread that not doing so created disadvantage. These individuals felt pressured to “pay-to-play”, i.e., to purchase loot boxes to stand a chance. These participants, who were all males but whose age and other demographics varied, were all competitive gamers (playing popular games such as FIFA and Call of Duty). Some participants described “paying-to-play” in a different sense, when coplayers or friends were “ahead” of them—and loot boxes helped catch up, to play alongside them. This links with social motives, discussed later.

#### 3.5.5. Enhanced Gameplay Experience

Almost all participants, regardless of their demographics and gaming preferences, reflected that loot boxes had currently, or in the past, enhanced their gaming experience. This encompassed both those who felt coerced into purchasing loot boxes, and those who did so willingly. Even when people were motivated strongly by the potential impact of box contents on their gameplay, they typically also reported the opening experience to be exciting and important to them; some explicitly described how the chance-based nature of the boxes enhanced their experience of the game itself (“it just makes the whole experience…a lot more interesting” (Sharon)).

#### 3.5.6. Investing in Games

Some participants reported supporting developers or investing in games as a motivating factor—particularly where the games were “free to play”, or by small/independent developers, encapsulating attitudes towards games and games developers, and dovetailing with the next, “social factors”, theme.

### 3.6. Social Factors

#### 3.6.1. Status/Esteem

Many participants who played collaborative and/or competitive games described the desire to enhance their status and/or esteem as an important motivator, and some (particularly males) spoke of “bragging rights” attached to successful openings, i.e., where rare, special or potent items are obtained, such as high-profile, high-performing “FIFA” players. As discussed under “value”, within games where character appearance is customisable (e.g., “Counter Strike”), skins from loot boxes can elevate status beyond rookie (“noob”), and some spoke not only of acceptance, but also admiration garnered when desirable items were won and displayed. Whilst participants aged from late-teens to mid-50s spoke of this, some felt that this motivation had particular significance for younger players, and many reflected on childhood experiences: (“It’s a status symbol…they can go into the playground and say ‘I’ve got this dance, have you got it? I’m better than you because I have this thing’” (Harry)).

#### 3.6.2. Influence of Friends/Other Players

Many participants reported an influence of friends/others on their decision to purchase a loot box. These tended to be the same participants who were motivated by socialising and/or desire for status/esteem, who were typically console and/or PC gamers, and were diverse in age and gender. Some described a relatively passive influence, where simply viewing friends or other players obtain desirable items motivated engagement—through feelings of envy or jealously. Some referred to a reluctant compliance with more direct peer pressure, and some reported feeling compelled by shame or mockery attached to not having loot box items. A desire to not stand out was also mentioned by some, because “you do kind of get targeted if you look like a noob—you will get all the other teams coming for you” (Mia)), highlighting a contrast between the positive attention reported when buying boxes vs. negative attention when not.

#### 3.6.3. Influence of Streamers and/or Professional Gamers

Many participants acknowledged that viewing streamers and/or professional gamers (on platforms like YouTube, Twitch or Discord) opening loot boxes had directly motivated them to follow suit, and some (with personal experience of streaming) felt that it influenced others. Some were acutely aware that “YouTubers spend thousands on packs” (Ian), but feared others may see the “highlight reel” of these opening sprees and assume they can replicate this by purchasing a small number of packs, or may even be motivated to purchase despite recognising the likely cost. Participants speaking about the influence of streamers/pro-gamers had varied demographic characteristics, but tended to be the same participants who reported other social motivation factors, and several were amongst those who had symptoms of problematic gaming and/or gambling, and those who spent the most money, spending between GBP 1000 and 4000 (ever) on loot boxes.

#### 3.6.4. Socialising

In contrast to social pressures, many participants described opening loot boxes as a means of socialising that they chose to participate in freely, where—either online or in person—peers gather for a shared opening experience. Here, purchasing loot boxes was driven by participatory, social and emotional factors. While the majority of socially motivated participants referenced both the positive (socialising) and the typically more negative (peer-influence) factors, a small number were motivated only by the former. These individuals tended not to be driven by game-progression, i.e., saw loot boxes as a way to socialise, rather than to compete within a game.

#### 3.6.5. Supporting Good Causes

Though very infrequently cited (by three of our participants), charity loot boxes, or specific items/events with donations provided to charities, are a relatively niche but existent phenomenon in videogames. Interestingly, all of these were predominantly solo gamers, who were not typically driven by other social motives.

### 3.7. Emotive/Impulsive Influences

Mood, emotion, and/or feelings of compulsion had motivated most participants’ purchasing at times, though most also reported the ability to resist these influences. Sometimes compulsive purchasing was contextualised as part of a general trait (“I’m very emotionally led” (Kate)), whereas for some it was linked to high emotional investment in a game or a desire to acquire characters/items that had emotional value.

#### 3.7.1. Urges, Temptation and/or Lack of Control

Most participants spoke of feeling compelled to purchase loot boxes to some extent, but the degree of compulsion varied. Some spoke of occasional impulse buying, whereby they just “felt like” it “on the day” (Kate), and some found it challenging to resist such feelings. Some described how their mood or state of mind influenced impulse-purchases; “if I’m feeling a bit spontaneous, a bit brash” (Susan). In more extreme cases, some spoke about feelings of “addiction”, most often when reflecting on historical purchasing—noting that they did not recognise their “addiction” at the time, but retrospectively feel that they were driven to make purchases by a compulsion, despite negative consequences (“I was kinda hooked” (Paul)). A small number of participants identified wider negative implications of loss of control over their purchasing (“it was getting out of hand” (Henry)) and wished to warn others against potential harms of loot boxes (“I don’t want people to have the same experience I did” (Dean)). Les felt loot boxes were “morally dubious”, and suggested that they “prey on people that do have…problems with addiction”.

Some enjoyed occasionally succumbing to the “temptation” to make a purchase, describing impulsive purchasing as a more positive experience, making comparisons with enjoyment of drinking alcohol in moderation, or playing the lottery (“it’s more, sort of, casual…like…one beer is not going to kill you” (Oscar)). However, these narratives tended to also highlight importance of knowing one’s limits, being able to enjoy the “buzz” in a controlled way (“I’m in control of those impulses most of the time, so I was able to say ‘okay, I’m not playing today’” (Zack)).

Interestingly, those who described feeling urges, temptation, or lack of control over their loot box purchasing were not consistently characterised by high scores on gaming (IGD) symptom scales—scores varied, with most in the 20′s on the IGD (below the ‘disordered gaming’ threshold), and only one scoring above threshold for problem gambling (although four did score above the moderate risk threshold), challenging the idea that “compulsive” loot box purchasers would typically be those reporting problematic gaming. There was a tendency for this motivation to be reported somewhat more consistently by problematic gamblers than by the sample as a whole, however, as suggested by Table 4.

#### 3.7.2. Boredom or Escapism

Some participants (particularly those who played across multiple platforms/styles) identified loot box engagement as a time-filling response to boredom—either in real life or within a game (“the game is getting a little bit stale…I’m a bit bored, I might think ‘oh I’ll do it’” (Zack)), with boredom sometimes described as precipitating urges or enhancing temptation, whereby people made unplanned purchases when under-stimulated. Boredom also connected to “escape” as a motivation. While some spoke of loot boxes as a way to escape from boredom, some also described them as a temporary escape from life, personal or social issues and large scale or day-to-day occurrences (“It’s like an escape, like, you get to go to another world” (Victoria)). Though often aligned to gaming more broadly, some participants identified the opening of loot boxes specifically as part of the escape process.

These boredom- and escapism-driven participants were diverse in age and gender, and contrary to what might be expected from the literature on escape-based coping being associated with addictive patterns of behaviour [17], they did not have strikingly high scores on the problem gambling or problem gaming scales, compared with the rest of the sample.

#### 3.7.3. Hard to Verbalise, Nonspecific Motivations

Some participants reported a lack of insight into, and/or ability to articulate their motivations (“I don’t really have an answer, just because” (Chris)), and although it is difficult to categorise such responses, we describe them under the broad theme of compulsive and emotive purchasing, as they share the quality of a lack of conscious/planned reasoning. It is important to recognise the existence of such instances where “nonspecific” or, perhaps, nonconscious motives are at play, because some participants described substantial expenditure despite a lack of insight into buying motivation.

### 3.8. Fear of Missing Out

A fear of missing out was frequently referenced as a driving factor; particularly the fear of missing out on shared social experiences. This has parallels with the construct of “FoMO”—identified as a frequent driver of social media engagement [35].

As discussed, there were social aspects to engagement for many, including “real world” events like parties or sleepovers, and some feared that if they did not buy loot boxes, they would be left out of these. One participant recalled buying loot boxes to avoid missing out on a shared in-game experience involving a distinctive skin that all their friends were wearing. Participants reporting fear of missing out socially tended to also report broader social motivations and were demographically diverse.

Whereas “FoMO” is most frequently used to refer to social media [35], our “fear of missing out theme” was broader than this, and also encompassed fear of missing out on time-limited events or offers. Some participants had bought many boxes out of fear they would miss their chance to get a coveted item—and this was pronounced amongst those who described being “collectors” of digital goods. This theme also encompassed feeling compelled to purchase loot boxes to get items needed to compete in “special events” (i.e., they feared missing out on participating if they didn’t get the special items). Those who feared missing out on promotions/events were markedly more likely to also report being driven by feelings of compulsion or urges, and there was a tendency for this motivation to be reported more consistently by those with problem gaming and/or gambling than those without, as can be seen in Table 4.

While this theme focuses on the anxiety about missing out that is generated through promotions and events, the promotions and events themselves (which also interact with other motivations such as desire for success or social participation) are better described as triggers or facilitators for purchase—discussed further, below.

### 3.9. Triggers/Facilitators

Most participants shared how game infrastructure triggered/facilitated loot box engagement. They described being driven by promotions (including time-limited items, and price-related offers/deals), special events in-game, and ease of purchase. None of these things in isolation would likely spur a player to purchase a box if they had no existing interest, and they were not the sole motivator for anyone, but they were potent in increasing the purchasing likelihood for players who had an underlying interest or motivation (i.e., the desire to obtain an item of value to them, which the promotion presents an increased opportunity for).

Many described being particularly susceptible to time-limited special offers related to seasonal events (such as Christmas-themed events in Overwatch or football-season-linked releases in FIFA), where they spent more than planned through desire to obtain something at the height of popularity or before it was “too late”. This echoes the “fear of missing out” theme, illustrating the interplay between facilitators and motivations. Beyond seasonal or annual events, some participants described how advertising (e.g., “teaser trailers”) kept them motivated.

Some spoke of being tempted to purchase a loot box by targeted advertisements or pop-ups that coincided with them being at an impasse within the game—illustrating how marketing and game design were carefully tailored to interact with players’ motivations such as the desire to progress.

Some commented that most devices can store payment details, enabling one-click purchases, and felt that ease and accessibility of purchasing made them more prone to buy without consideration (reflecting the “emotional/impulsive” theme). Some felt their spending divorced from real money, due to lack of physical signifiers like a card/cash. While parallels between loot boxes and gambling were often made, some noted that buying loot boxes was “easier” and less constrained than gambling—facilitating heavy engagement.

## 4. Discussion

This in-depth qualitative study affords novel insights into reasons and facilitators for loot box purchasing, derived from those with lived experience, and summarised under seven broad (interlinked and overlapping) themes: opening-related factors; value of items; game-related factors; social factors; fear of missing out; compulsive/emotive factors; facilitators.

That the act of opening loot boxes was psychologically and emotionally rewarding, with the “reveal” creating excitement, mirrors other phenomena within digitised consumer cultures. The popularity of YouTube “unboxing” videos [36], for instance, illustrates how the “revealed” object’s utility can be secondary to the enjoyment of the reveal, (echoing the idea that the desire for an object is sometimes more alluring than possessing the item itself; [37,38]). Frequent “refreshing” of available items by game developers stimulates this; participants reflected that the chance of winning novel items garnered desire and encouraged further purchases. That participants found “near misses’” (where colours, sounds or animations hint at high-value items that do not materialise) stimulating, supports the idea that anticipation is rewarding in itself, mirroring traditional gambling, where near misses are highly motivating [39].

That excitement of opening boxes was linked with anticipated content value also mirrors gambling, where the outcome’s (monetary) value is instrumental in generating excitement [40], and indeed, is consistent with findings from Larche and colleagues that finding rare items in loot boxes generates physiological arousal [41]. In contrast to gambling, though, the value of loot box contents was often subjective and mediated by a range of factors. Some judged value in monetary terms (by items’ outright purchase or trade-in value), while others’ judgements were influenced by cultural factors and/or the gratifications they were seeking through gaming and/or purchasing loot boxes. For example, functional items were highly valued by those wanting to progress in-game and/or beat others, linking with both game-related and social (competition) themes. Items holding purely cosmetic value tended to be important for those who were socially motivated and wanted to attract attention, gain social approval or avoid stigma, and those who felt good about themselves when their avatar “looked good”. These values and motivations align with theoretical frameworks based around need satisfaction that have been applied to motivations for both gaming [42] and gambling [43]—such as self-determination theory’s assertion that motivations reflect a desire for competence, autonomy and relatedness [44]. A desire for relatedness could explain the social motivations, while desire for competence and autonomy could explain many of the game-related motivations, and the valuing of functional items.

Some motivations were associated with intentional decisions to buy, grounded in a positive want or desire—e.g., to enhance game enjoyment. Others were more negatively framed, and associated with a perceived need or compulsion, or a reaction to boredom or craving. This parallels the idea that video gaming is motivated by both “push” factors (e.g., positive gratification of need satisfaction that can be attained in-game), and “pull” factors (e.g., need frustration in one’s real-world life) [40]. This highlights the importance of considering the deeper underlying motivations to gain a full understanding of why people engage with loot boxes, because a similar “surface” reason (e.g., seeking functional items to boost performance) could be driven by different factors for different individuals. Some bought loot boxes when feeling a need for a boost (e.g., to compensate for negative emotions after a harsh defeat), for instance, whereas others chose (less emotively) to make purchases to positively enhance their gaming experience. Self-determination theory again has relevance, here—in wider gaming research, those with low “real-life” need-satisfaction were more prone to engage problematically with videogames (e.g., to fulfil the need for competence), but gamers high in real-life need satisfaction could still enjoy frequent gaming and its positive impact on sense of competence [45].

It was unsurprising that boredom motivated the purchasing of loot boxes. Increasingly ubiquitous digital and gaming infrastructures fill hitherto brief interludes of free time (e.g., the commute and the lunch break), to the point where Hand [46] postulates that they impede the “profound” boredom that encourages self-reflection [47], replacing it with “delusional escape” into “digital boredom” instead—characterised by fragmentation, repetition and standardisation [46]. This aligns with some participants’ accounts of how their engagement with loot boxes was driven by boredom engendered by the game itself (e.g., when a level or task became repetitious, but could be skipped or completed with the help of the contents of a loot box)—again illustrating how game features can nudge players towards purchases.

It was notable that, despite previously established links between loot box engagement and other behavioural addictions [48], several participants reported neither disordered gaming nor problematic gambling symptoms, yet described their loot box purchasing as motivated by “temptation” or “compulsion”, and struggled with controlling it, i.e., some gamers experience risky loot box purchasing without co-occurring problems with gambling or gaming. It has been argued that increasingly digitalised forms of “repeat play” gambling [49] may be characterised by different symptoms than those of traditional gambling [16], where “addiction” is driven not by pursuit of excitement, but by desire for an affective state of being “in the zone” or in a state of flow. If this translates to loot box purchasing, it might explain why some people demonstrating problematic engagement are not prone to over involvement in traditional gambling.

Some participants reflected on the importance of externally situated factors, such as time-limited offers that generated a “fear of missing out”; points in a game that were almost impossible to progress past without the contents of purchased boxes; games where box purchasing was so prevalent that one could not be competitive without participating in it. These factors typically combined with other (internally located) motivations (e.g., desire to progress or be competitive) to influence behaviour. Such external influences often impacted people who reported very little intrinsic desire to engage with loot boxes. Contrasting with those who actively enjoyed purchasing them as an enhancement to their gaming, some players whose primary interest was gaming purchased loot boxes reluctantly, as a “means to an end”. Notably, while we asked participants to focus on their loot box purchasing motives, they frequently digressed into discussing broader gaming motivations, which were usually closely connected. This is consistent with the assertion that understanding loot box engagement requires understanding of wider gaming involvement [18], and with the finding that loot box purchasing is often correlated with both problematic gaming and problem gambling [3,44].

Furthermore, a recent study found loot box spending was correlated with peer spending behaviour, rather than with measures of gaming “addiction” or involvement [50], suggesting that in certain demographics (or players of particular games), social factors may, in fact, put people at higher risk of overspend than “addiction” to either gaming or gambling. This is consistent with some of our participants’ accounts of how important social factors were in their loot box engagement, and also parallels observations that sectors of the gambling industry have become increasingly socialised and embedded within wider leisure experiences and friendship networks. Rayman and Smith [51] note how sports betting, situated within the wider masculine weekend leisure experience of sports fandom and the night-time economy, led many participants with no previous history of gambling to develop problematic spending habits to preserve their “liquid friendships”, group memberships and identities. There are parallels to be further explored here around identity, gaming, and loot box overspending within the context of late-modern digital culture, in which identity, self-worth and access to friendships are often tenuously organised around particular leisure pursuits [52].

Our work is largely novel, particularly considering the depth of detail and expansion provided throughout our work, however, the foundations for such work were established by Zendle, Meyer and Over [24]. As part of a primarily quantitative study of loot box engagement in just under 500 adolescents, they uncovered a series of motivations—drawn from free-text responses to an open-ended online survey question. These findings and our study are complementary; where they had a large number of participants but relatively little depth of detail (due to the inevitable constraints of an online survey for qualitative work, meaning only brief responses to a single question were collected), the current study focused on a smaller sample, allowing expansion, exploration and development of these ideas through in-depth one-to-one interviews. The motives noted by Zendle, Meyer and over were gameplay advantages; seeking specific items and characters and to create a collection; fun, excitement and thrills of the box opening; cosmetic reasons; supporting developers or paying for the game; perceptions that loot boxes are good value; time advantages and profit. All of these ideas emerged unprompted in some form within our interviews and are represented within our own expanded themes, but we were able to provide greater depth of insight into the nuances of these motivations, how they vary between people, and how they interact. For example, whereas Zendle, Meyer and Over identified “cosmetic reasons” as a broad motivation [24], we learned in more depth about how the appearance of a gamer’s avatar could be important to them for a range of social reasons, boosting their self-image or social standing. Like Zendle, Meyer and Over, we found gameplay advantages to be a prominent theme, but also learned how this driver can vary across platforms and game types, and how certain types of gamer (often males who engage in frequent and varied gaming) are particularly concerned with gameplay advantages. We also uncovered opposing attitudes towards loot boxes containing items that confer such advantages. Furthermore, we identified several motivations that were not reported in [24] but were significant driving factors for some of our participants, including social motivations, fear of missing out, and compulsive/emotive themes.

### 4.1. Strengths and Limitations

The diverse combinations of motivations that influenced engagement with loot boxes highlights why our use of an in-depth qualitative methodology was a particular strength, supporting a holistic understanding of the complex array of influences that can interact to drive purchasing.

Another strength of the current study is the size and heterogeneity of the sample. We exceeded the 25 participants deemed an acceptable minimum for in-depth interviews [53], and participants were from a wide geographical spread across the UK, with good diversity of demographic characteristics (including ethnicities, ages, income, employment and living situation). The sample was around 60% male, but this reflects the fact that gaming is still typically identified as a somewhat more heavily male-oriented pastime [54].

While all participants had experience of buying loot boxes, and engagement varied considerably across our sample, only a few might be described as “whales” (very high spenders on gaming microtransactions/in-app purchases [6,55]). This reflects the fact that very high levels of expenditure are only engaged in by a small percentage of loot box buyers [5,6] but further work with a larger sample of “whales” would be of value.

### 4.2. Implications and Future Research

A major contribution of this study is the insight it provides into the way gamers evaluate the worth of in-box contents, and into how this can drive purchasing. Debates around legislation have tended to prioritise the question of whether items won have monetary value, e.g., through re-sale, and recent findings suggest that they often do meet this criterion [56]. However, in addition to providing additional evidence that some gamers are directly motivated by the desire to sell on the contents of a loot box for in game and/or real-world currency, our study also illustrates how contents can also hold significant social or psychological value (often tied to gaming involvement) outside the narrow “monetary worth” definition. In other words, items within a loot box can be extremely alluring and psychologically rewarding, and can generate high levels of excitement, even when their cash-out value per se is not a consideration for the buyer. This has important implications for measuring and/or preventing harm in excessive loot box purchasers, because the fact that gaming involvement appears to mediate the value of items to players may mean “symptoms” of pathological engagement diverge from those seen in traditional gambling, and that prevention measures need careful tailoring [18].

This in-depth qualitative study will inform the development and validation of a scale to quantitatively measure the drivers of loot box purchasing. This can then be utilised in a large-scale survey to identify, amongst other things, whether there are significant differences in the patterns of motivations reported by those with symptoms of problematic gaming, gambling, or loot box engagement, and those who do not experience such difficulties. Within the current sample, there are some indications that, while problematic gamers and gamblers are driven by many of the same motives as those without problems, they seem particularly prone to being motivated by feelings of compulsion, to being influenced by streamers and professional gamers, and to being afraid of missing out on promotional offers, but quantitative data from a large, diverse sample is needed to confirm whether these patterns are seen more widely and consistently. If so, there are potential implications for policy and education; limiting the coverage of loot box openings in videos by professional gamers and streamers; restrictions on game developers’ use of time-limited offers—or at least on the targeting of these to vulnerable (high spending) individuals, for instance, may be recommended.

## 5. Conclusions

This study provided an in-depth account of diverse factors that motivate people to buy loot boxes, which can help academics, clinicians and policymakers to understand how and why people engage with chance based in-game mechanics. It will also feed into the development and validation of a formal scale, to quantitatively measure self-reported reasons and facilitators for loot box engagement. Based on the gambling and gaming literature, we expect this will assist with identification of people at greater risk of developing problematic loot box involvement, with important implications for prevention of harm, for example, through educational messaging.

## Figures and Tables

**Table 1:** Sample characteristics: demographics, gaming and gambling symptom scores, and loot box spend.

Pseudonym	Age	Gender	Ethnicity	Geography	Education	Marital	Living	Employment	Individual Salary (GBP)	IGD	PGSI	Monthly Spend	Yearly Spend	All Time Spend
Alex	22	M	White—British	East Mids England	UG higher education	Single	With parents	FT employment	25,001–30,000	21	1	GBP 20	x^3^	GBP 700
Andrew	20	M	White—British	North East England	UG higher education	Single	With parents	PT employment	<10,000	x	x	x	GBP 1000	x
Charlie	46	M	White—British	West Mids England	UG higher education	Divorced	Partner/children	Self employed	40,000+	22	0	0	GBP 4	GBP 50
Chris	25	M	Gypsy/Irish Traveller	South East Wales	Secondary school	Married	Partner/children	FT employment	20,001–25,000	25	2	GBP 50	GBP 150	GBP 3000
Daniel	26	M	White—British	West Mids England	College/vocational	Cohabiting	Partner/children	FT furloughed	25,001–30,000	16	3	GBP 50	GBP 300–500	x
Darren	31	M	White—British	East Mids England	Secondary school	Cohabiting	Partner/children	FT employment	10,000–15,000	16	4	GBP 150	GBP 1000	GBP 7000
Dean	26	M	White—British	South West England	UG higher education	Cohabiting	Partner/children	FT furloughed	20,001–25,000	34	10	x	GBP 2000	GBP 4000
Debbie	29	F	Black—African	South East England	PG masters	Cohabiting	Partner/children	FT employment	30,001–40,000	19	0	GBP 4	GBP 20	GBP 200
Emily	19	F	White—British	North East England	College/vocational	Cohabiting	Partner/children	Seeking opportunities	Below 10,000	11	0	GBP < 10	GBP 50–100	GBP 200
Harry	24	M	White—British	Highlands—Scotland	UG higher education	Single	Sharing property with non-family	FT employment	25,001–30,000	26	0	x	x	GBP 20
Henry	18	M	White—British	South East England	College/vocational	Single	With parents	Seeking opportunities	Not earning	26	0	GBP 40	x	x
Ian	22	M	White—British	South West England	UG higher education	Single	Student housing	FT education	Not earning	29	8	GBP 100	GBP 300	GBP 4000
Kate	35	F	White—British	South East England	UG higher education	Cohabiting	With partner	Self employed	<10,000	14	0	GBP < 10	GBP 50	GBP 100
Les	28	M	White—British	South Wales	UG higher education	Single	Sharing property with non-family	FT employment	30,001–40,000	22	0	GBP 4	GBP 50	GBP 300
Mia	18	F	White—British	South West England	College/vocational	Single	With parents	FT education	Not earning	31	0	GBP 30	x	x
Natalie	56	F	White—British	South East England	UG higher education	Prefer not to say	Living alone	Living with disability	Not earning	15	0	x	GBP 100	GBP 100
Neil	44	M	White—British	South West England	College/vocational	Cohabiting	Partner/children	FT employment	Above 40,000	18	18	GBP 25	GBP 300	GBP 1200
Oscar	34	M	White—British	South West Wales	PG masters	In a relationship	Partner/children	FT furloughed	20,001–25,000	19	0	GBP 3.50	GBP 40	GBP 160
Paul	40	M	White—British	North West England	College/vocational	Married	Partner/children	FT employment	30,001–40,000	22	4	GBP 60	GBP 700	GBP 3000
Roger	18	M	White—British	South East England	College/vocational	Single	With parents	PT furloughed	10,000–15,000	20	4	x	x	GBP 1000
Sarah	29	F	White—British	North East England	College/vocational	Married	Partner/children	PT employment	<10,000	18	0	x	x	GBP 15
Seb	21	M	White—British	North East Scotland	Secondary school	Single	Living alone	FT education	Not earning	20	0	x	x	GBP 250
Sharon	24	F	Chinese	South East England	PG masters	Single	With parents	Other	N/A	24	0	x	GBP 30	GBP 100
Spencer	28	M	White—Eastern European	North West England	UG higher education	Single	Alone	FT employment	40,001+	20	0	0	0	GBP 50–60
Susan	22	F	White—British	West Mids England	UG higher education	In a relationship	With parents	FT education	Not earning	22	0	x	GBP 30	GBP 250
Tom	29	M	White—British	North West England	UG higher education	Single	Living alone	Other	Below 10,000	15	0	GBP 2.50	x	GBP 30
Victoria	29	F	White—British	South West England	College/vocational	Married	Partner/children	FT employment	30,001–40,000	20	0	GBP 20–50	GBP 240–600	x
Zack	29	M	White—British	South West England	PG masters	Cohabiting	Partner/children	FT employment	15,001–20,000	14	2	GBP 20–80	GBP 100	GBP 300

Internet Gaming Disorder Scale IGD-SF9 [29]; Problem Gambling Severity Index (PGSI) [28]; “x” refers to an answer not known or given.

**Table 2:** Illustrative quotations by theme.

Opening Experience			
Box-Related Factors		Opener-Related Factors	
“if I buy a loot box now, they definitely make it exciting to do this…there’s a lot of animation that comes with it and that’s quite exciting and thrilling.” (Susan) “It’s, like, a walkout scene, so each player it would be, like, “striker! Left” or “striker, Portugal” and it will start to show the cards after, six seconds of when you opened the pack…It’s definitely become more addictive” (Ian)		“It was fun, you got what you wanted or you didn’t; it was still all good fun.” (Natalie) “Just like a rush…a rush of excitement…just pleasure, really, it was like a hit…Especially if you got a good player, like, a rare player. It was just, like ultimately winning” (Sharon)	
**Value of Box Contents**			
**Financial**	**Aesthetic/Cosmetic**		**Functional**
“If you got a good player…it was, like, ultimately winning virtual currency, because you could sell that player for virtual currency, so that’s what it was all about.” (Sharon) “If I put in a load of money in at the start, I’m going to create a lot more money for the future.” (Ian)	“It’s just an opportunity for you to buy the skin and buy something that you think looks good” (Les) “there was quite a lot of in-game shame for people who just have the default skins on weapons and characters.” (Mia)		“I sit here and think how much am I going to use this thing” (Spencer) “it’s not so much for display, but for advancement, for me” (Susan)
**Game-Related Elements**			
**Progression**	**Skip the Grind**		**Pay to Win**
“I play some of the puzzle games, mainly on my phone…and sometimes if a level’s been driving me bonkers for ages and I’m one move away, and I’ve run out of lives, I’ll pay a pound for an extra life.” (Kate)	“You can either spend a lot of time grinding it for free or you can, like, cheat, well—not cheat, but shortcut your way in by just spending money and just getting the content as well” (Sharon)		“just wanting to be able to do better, so, in the games where it give you items, and, so, you get that special item that will help you out…beat that last boss, or help beat more people online.” (Paul)
**Pay to Play**	**Enhanced Game Experience**		**Investing in Games**
“I don’t like it but it’s a necessity, for the sake of me being able to play” (Roger) “if you don’t buy packs or you don’t’ grind the game for hours…it’s just not possible to be competitive.” (Oscar) “if the rest of my team are quite far ahead within a game and I need to catch up to that point…I would fork out.” (Emily)	“I had a lot of fun playing the game…having these load outs, from the loot box were affecting the gameplay, giving me new weapons, making my characters more stronger…made it more fun.” (Harry)		“I like to give back to the developers of it if it’s something that I think looks cool or I’m kind of interested in.” (Tom) “Most of these games that offer them are free to play, so others, some people justify the purchase, saying this game gives me entertainment, so I’m going to pay for it.” (Roger)
**Social Factors**			
**Status and Esteem**	**Influence of Friends/Others**		**Influence of Streamers and/or Pro-Gamers**
“You could brag to the lads at work, “I just packed so and so in a pack last night…” (Darren) “It was very important to get those achievements and to get these limited-edition items that no one else had, it was kinda like a status thing…in these types of games, you were put higher on the social ranks if you could display these skins…“oh look at everything that I’ve got,” you know? There’s that power that comes behind with it.” (Susan)	“It might be that my friend Gerard gets a really cool skin, and I’m like “well, now I want it”, or, I’m then comparing myself to him, because he’s got it and I don’t” (Zack) “everybody else was doing it, like, ‘ah, yeah you haven’t got it’… I’d probably give in to peer pressure” (Chris) “if you have a default skin, a default load out…they’ll just be rude to you…to get some more respect in the game you do have to have, skins and stuff, but it’s another motivation.” (Mia)		“The influence online is crazy, if there wasn’t influence, I don’t think there would be more sales of loot boxes…” (Ian) “You look at some of the reactions on YouTube and it’s like; if you pull a good player, people go absolutely crazy, like, ‘YES! YES! YES!’ because you pulled that amazing item” (Ian)
**Socialising**	**To Support Good Causes**		
“I’d be out with my friends a few of us would all normally play FIFA and we’d be like “oh, actually shall we all just throw like a tenner on some packs?”…see what we can get.” (Oscar) “If I’m opening a loot box and there’s other people that I’m chatting to and they’re opening loot boxes, and you can, it’s a shared experience, they’re, like “ah, great you go that you wanted”, you know, or “ah, sorry about that—maybe next time” and it’s the same, you’re the same with them, it’s a kind of camaraderie, almost, like disappointment on a social scale or happiness on a social scale.” (Natalie)	“They do charity events once a year, or a couple of times a year, where it says like ‘spend GBP 10 and you will get this rideable mount’ and you just move around on it, you fly around on it, and it looks special, and all the money will go to charity…the money goes to a cause” (Roger)		
**Emotive/Impulsive Motivations**			
**Urges, Temptation and/or Lack of Control**	**Boredom or Escapism**		**Hard to Verbalise, Non-Specific Motivations**
“it was always very difficult to resist the temptation” (Seb) “I realised that was an addiction but then it kept slipping my mind and every time it slipped my mind it sort of got replaced with ‘oh when can I buy more, when do I get more money, when can I buy more’” (Neil)	“Sometimes you sit there, and you think, ‘well, hold on, I’m a little bit bored, I don’t really want to watch TV, I know, I’ll open some FIFA packs, and buy some games add-ons’ and, you know, I’m sure I’m not the first person to say ‘well, I’m just bored…I’ll put money on needlessly’” (Darren) ‘		“Well, why I did, that’s a tough one isn’t it, the why is probably just the, I don’t know” (Spencer) “I don’t know, really—it’s a bit embarrassing in a group of 20-year-olds, 21-year-olds now, you know, like, to be sitting there putting hundreds of pounds in to what is a football game on Xbox.” (Sharon)
**Fear of Missing Out**			
“fear of missing out, that’s the, that’s what people are most vulnerable to—especially if they’re just getting in to a game and they think ‘oh wow, I want to really get into this and do well in this game’ or something, and then they put a time limited event on and you think ‘hang on a minute...maybe I need to buy something’” (Sharon)			
**Triggers/Facilitators**			
**Promotions**	**Special (Time-Limited) Events**		**Ease of Purchase**
“…they would give you, like, 20% extra free if you spent GBP 80 straight up, as opposed to just 20, or they give you a better pack with more chance of getting a good player if you spent more money on the game, so more money on the pack.” (Sharon)	“they would have this time-limited event going on, which brought the rate up and a lot of people… would end up resorting to buying, additional tickets to try and roll for the unit they want” (Sharon) “the advertising is so good…that’s why you continue to put money in, and money in” (Ian)		“you could link a card to your account…it doesn’t feel like you’re spending money…you’re not seeing any money exchange hands.” (Paul) “When you’re gambling online, you have to go through the whole system of signing up, and confirming…on PS4 it’s like, buy, done…I could spend GBP 500 in five seconds.” (Ian)

**Table 3:** Distribution of themes across the sample for opening experience, value of content, game related and social influences.

Theme	Opening Experience		Value of Content			Game Related						Social Influences				
Pseudonym	Box Related	Opener Related	Aesthetic /Cosmetic	Functional	Financial	Pay to Win	Pay to Play	Progression	Skip the Grind	Improve Game Play	Invest in Game	Status and Esteem	Friends/Others Do It	To Socialise	Streamers/Pro Gamers	Good Causes
Alex																
Andrew																
Charlie																
Chris																
Debbie															
Emily																
Harry																
Henry																
Kate																
Les																
Mia																
Natalie																
Oscar																
Sarah																
Seb																
Sharon																
Spencer																
Susan																
Tom																
Victoria																
Zack																
Daniel																
Darren																
Paul																
Roger																
Ian																
Neil																
Dean																
Total	21	23	22	23	9	6	11	9	8	24	4	14	12	12	12	3
Amount	Most	Most	Most	Most	Many	Some	Many	Many	Some	Most	Some	Many	Many	Many	Many	Some

Key: “some” = 1–8 participants; “many” = 9–18 participants; “most” = 19–28 participants. Coloured cells mean that a theme was endorsed. Green = participants below cutoffs for problematic gaming and gambling. Blue = participants scoring 3+ on PGSI (i.e., those with “at risk” gambling). Red = participants scoring 8+ on PGSI (i.e., “problem gamblers”). Purple = participants scoring both 32+ on IGD and 8+ on PGSI (NB: there were no participants scoring 32 or above on IGD who did not also score 3 or above on PGSI). Darker-shaded cells = a participant spoke about a motivation in general terms (i.e., as something that they thought motivated others rather than endorsing it as a personal motivation.

**Table 4:** Distribution of themes across the sample for emotive/impulsive influences, fear of missing out, type of gamer and style of gaming.

Theme	Emotive/Impulsive Influences			Fear of Missing Out		Type of Gamer			Style of Gaming		
Pseudonym	Urges/Temptation/Control	Boredom or Escape	Hard to Verbalise/Nonspecific	Missing Out on Social Experience	Missing Out on Time offers/Promotions	Mobile	PC	Console	Cooperative	Competitive	Solo
Alex											
Andrew											
Charlie											
Chris											
Debbie											
Emily											
Harry											
Henry											
Kate											
Les											
Mia											
Natalie											
Oscar											
Sarah											
Seb											
Sharon											
Spencer											
Susan											
Tom											
Victoria											
Zack											
Daniel											
Darren											
Paul											
Roger											
Ian											
Neil											
Dean											
Total	19	6	4	12	18	22	15	23	15	22	22
Amount	Most	Some	Some	Many	Many	Most	Many	Most	Many	Most	Most

Key: “some” = 1–8 participants; “many” = 9–18 participants; “most” = 19–28 participants. Coloured cells mean that a theme was endorsed. Green = participants below cutoffs for problematic gaming and gambling. Blue = participants scoring 3+ on PGSI (i.e., those with “at risk” gambling). Red = participants scoring 8+ on PGSI (i.e., “problem gamblers”). Purple = participants scoring both 32+ on IGD and 8+ on PGSI (NB: there were no participants scoring 32 or above on IGD who did not also score 3 or above on PGSI). Darker-shaded cells = a participant spoke about a motivation in general terms (i.e., as something that they thought motivated others rather than endorsing it as a personal motivation).

## Data Availability

The data for this study are not publicly available beyond anonymous quotations as cited throughout—this is in accordance with the restriction and boundaries of the ethical approval received.
